# Antimicrobial Resistance of *Enterococcus* sp. Isolated from Sheep and Goat Cheeses

**DOI:** 10.3390/foods10081844

**Published:** 2021-08-10

**Authors:** Jana Výrostková, Ivana Regecová, Eva Dudriková, Slavomír Marcinčák, Mária Vargová, Mariana Kováčová, Jana Maľová

**Affiliations:** 1 Department of Food Hygiene Technology and Safety, University of Veterinary Medicine and Pharmacy in Košice, Komenského 73, 041 81 Košice, Slovakia; jana.vyrostkova@uvlf.sk (J.V.); eva.dudrikova@uvlf.sk (E.D.); slavomir.marcincak@uvlf.sk (S.M.); mariana.kovacova@student.uvlf.sk (M.K.); jana.malova@uvlf.sk (J.M.); 2 Department of Public Veterinary Medicine and Animal Welfare, University of Veterinary Medicine and Pharmacy in Košice, Komenského 73, 041 81 Košice, Slovakia; maria.vargova@uvlf.sk

**Keywords:** antimicrobial resistance, cheese, *Enterococcus* sp., MALDI-TOF, PCR

## Abstract

This study aimed to calculate the proportion of antibiotic resistance profiles of *Enterococcus faecium*, *E. faecalis,* and *E. durans* isolated from traditional sheep and goat cheeses obtained from a selected border area of Slovakia with Hungary (region Slanské vrchy). A total of 110 *Enterococcus* sp. were isolated from cheese samples, of which 52 strains (*E. faecium* (12), *E. faecalis* (28), *E. durans* (12)) were represented. After isolation and identification by polymerase chain reaction and matrix-assisted laser desorption/ionization-time-of-flight mass spectrometry, the enterococci (*E. faecium, E. faecalis*, and *E. durans*) were submitted to susceptibility tests against nine antimicrobial agents. In general, strains of *E. faecalis* were more resistant than *E. durans* and *E. faecium*. A high percentage of resistance was noted in *E. faecalis* to rifampicin (100%), vancomycin (85.7%), teicoplanin (71.4%), erythromycin (71.4%), minocycline (57.1%), nitrofurantoin (57.1%), ciprofloxacin (14.3%), and levofloxacin (14.3%). *E. durans* showed resistance to rifampicin (100%), teicoplanin (100%), vancomycin (66.7%), erythromycin (66.7%), nitrofurantoin (66.7%), and minocycline (33.3%), and *E. faecium* showed resistance to vancomycin, teicoplanin, and erythromycin (100%). Multidrug-resistant strains were confirmed in 80% of the 52 strains in this study. Continuous identification of *Enterococcus* sp. and monitoring of their incidence and emerging antibiotic resistance is important in order to prevent a potential risk to public health caused by the contamination of milk and other dairy products, such as cheeses, made on farm level.

## 1. Introduction

Enterococci are Gram-positive bacteria belonging to the lactic acid bacteria (LAB) group. They occur mainly in the human intestine. They are commensal microorganisms, although over the last 20 years, due to the acquisition of antimicrobial resistance, they have become important hospital-acquired pathogens [1]. Nevertheless, they still have an important role in the production of fermented dairy products, such as cheeses, in prolonging their shelf life and improving their organoleptic properties [2,3].

However, the occurrence of enterococci in food is also an indicator of poor manufacturing practice and product contamination, as they are commonly present in raw milk, stressing the importance of focusing on both the raw materials used in cheese production and the products they encounter during production [4,5,6].

In addition to their function as indicators of poor manufacturing practice, enterococci have a technological function in the production of fermented products. However, they may exhibit different levels of antibiotic resistance, which is one of the main concerns of these food isolates [7,8,9]. Due to this ability, enterococci exhibit a wide repertoire of antibiotic resistance mechanisms. Enterococci can become the dominant flora in the gastrointestinal tract under antibiotic pressure, predisposing a severely ill and immunocompromised patient to invasive infections [10,11].

Therefore, they have become recognized as important nosocomial pathogens due to their natural intrinsic resistance to several antimicrobials, as well as their ability to rapidly acquire virulence and determinants of multidrug resistance [12,13].

Antimicrobial resistance is one of the critical public health problems, so it needs to be monitored in bacterial strains in various environments as a strategy to combat resistant bacteria [14].

Based on the above-mentioned considerations, the aim of our study was to detect the presence of *Enterococcus* sp. from sheep and goat cheese produced on a farm located in the border area of Slovakia with Hungary and, subsequently, determine the antimicrobial resistance of bacteria found in these products. The cheeses tested by us belong to the group of so-called RTE (ready-to-it) cheeses. We also include bryndza among such products. Bryndza is a typical Slovak cheese made from raw milk with no special starter culture [15]. 

Although there have been numerous studies on antimicrobial resistance, there is not currently enough information from local studies on produced milk as well as products from milk at farm level in selected parts of the studied territory. In addition, most studies have addressed the prevalence of antimicrobial resistance in enterococci in cow’s milk products, but not the antimicrobial resistance of enterococci in sheep’s and goat’s milk products. This study contributes to the acquisition of knowledge in this area.

## 2. Materials and Methods

### 2.1. Isolation and Identification of Strains

From May to September 2020, we isolated enterococcal strains from sheep (*n* = 10) and goat (*n* = 10) cheese samples. The cheeses were made from unpasteurized milk without the addition of an initial cheese culture and matured for 30 days. Subsequently, a stock suspension and decimal dilutions were prepared from all tested samples according to ISO norm [16].

Enterococcal isolates from the examined samples were isolated according to Koreňová et al. [17]. Subsequently, based on the characteristic appearance, typical colonies of bacteria were used for identification by PCR reaction. 

The DNA was isolated from enterococcal strains according Hein et al. [18]. The PCR method was used to identify the genus of *Enterococcus* according to Ke et al. [19] and Martineau et al. [20]. As an internal control for PCR, primer sequences derived from the bacterial *16S rRNA* gene and primers derived from the *tuf* gene were used to obtain a specific sequence for the genus *Enterococcus* sp. Each primer was synthesized in Generi Biotech s.r.o. (Table S1; Hradec Králové, Czech Republic).

The PCR protocol was optimized as follows: initial denaturation at 95 °C for 12 min, with the next step including 30 cycles (denaturation at 95 °C for 20 s, annealing at 55 °C for 60 s, extension at 72 °C for 2 min). Final extension was performed at 72 °C for 10 min. The HotFirepol^®^ Mastermix (Ecoli s.r.o., Bratislava, Slovakia) was used in the PCR reaction. PCR products were visualized in agarose gel with Goldview™ Nucleic acid stain (Beijing SBS Genetech Co. LTD., Beijing, China) by using the MiniBIS Pro^®^, (DNR Bio-Imaging system Ltd., Neve Yamin, Israel). 

Identification using MALDI-TOF MS was performed using Flex Analysis software, version 3.0 on an Ultraflex III instrument and BioTyper software, version 1.1 (Bruker Daltonics, Billerica, MA, USA). For the most accurate analysis, individual samples were prepared by an extraction procedure using ethanol and formic acid [21]. The samples were analyzed in cooperation with the Institute of Animal Physiology of the Slovak Academy of Sciences in Košice.

### 2.2. Assessment of Antibiotic Sensitivity 

The susceptibility of isolated bacterial strains to selected antibiotics and multi-drug resistance (MDR) was determined by the agar dilution method (ADM) according to the procedure described by CLSI document [22]. MDR was defined as acquired non-susceptibility to at least one agent in three or more antimicrobial categories [22]. ADM was performed on Petri dishes with Müeller-Hinton agar (Hi-Media, Mumbai, India) in duplicate. Test plates containing different concentrations of antibiotics were used for the determination of minimum inhibitory concentrations (MICs). After inoculation, these plates were incubated at 37 °C for 24 h. Detected MICs were evaluated according to CLSI document [22].

In determining the MICs, assay plates were used with the following final concentrations of antibiotics on *Enterococcus* sp.: ciprofloxacin (CIP) 0.5–8.0 mg/L; doxycycline (DO) 2.0–32.0 mg/L; erythromycin (E) 0.25–16.0 mg/L; nitrofurantoin (F) 16.0–256.0 mg/L; levofloxacin (L) 1.0–16.0 mg/L; minocycline (MH) 2.0–32.0 mg/L; rifampicin (RD) 0.5–8.0 mg/L; teicoplanin (TEC) 4.0–64.0 mg/L; vancomycin (VAN) 2.0–64.0 mg/L.

MIC breakpoints to determine antibiotic resistance were as follows [22]: CIP ≥ 4 mg/L; DO ≥ 16 mg/L; E ≥ 8 mg/L; F ≥ 128 mg/L; L ≥ 8 mg/L; MH ≥ 16 mg/L; RD ≥ 4 mg/L; TEC ≥ 32 mg/L; VAN ≥ 32 mg/L. 

After detecting the phenotypic manifestation of antimicrobial resistance in enterococci, the following genetic determinants of resistance were detected: *vanA*, *ermA*, *ermB, ermC*, and *msrC* (Table S1). The PCR reaction conditions were the same (except for the annealing temperature; Table S1) for the genus identification of the isolates above. The obtained gene sequences from the studied strains used in this work were submitted to the GenBank-EMBL database. The obtained DNA sequences were searched for homology to those available at the GenBank-EMBL database using the BLAST program (NCBI software package).

*Enterococcus* (*E*.) *faecalis* CCM 4224 (Czech Collection of Microorganisms, Brno, Czech Republic) was used in this study as a reference strain for the PCR method and ADM.

### 2.3. Statistical Analysis 

Significant difference analysis of isolates of *Enterococcus* sp. antibiotic resistance was made by the odds ratio (OR) test conducted with MedCalc Statistical Software version 19.2.6 (MedCalc Software, Ostend, Belgium) according to Regecová et al. [23]. A confidence interval was set to *p* < 0.05 at 95%. 

## 3. Results

By microbiological culture examination of individual cheese samples and subsequent identification of isolates by PCR method, 110 isolates of *Enterococcus* sp. were obtained. Subsequently, we performed species identification of the 110 examined isolates by MALDI-TOF MS method; we identified 3 bacterial species, namely, *E. faecium* (12 isolates: goat cheese–8 isolates; sheep cheese–4 isolates), *E. faecalis* (28 isolates: goat cheese–13 isolates; sheep cheese–15 isolates), *E. durans* (12 isolates: goat cheese–8 isolates; sheep cheese–4 isolates). 

**Figure 1:**
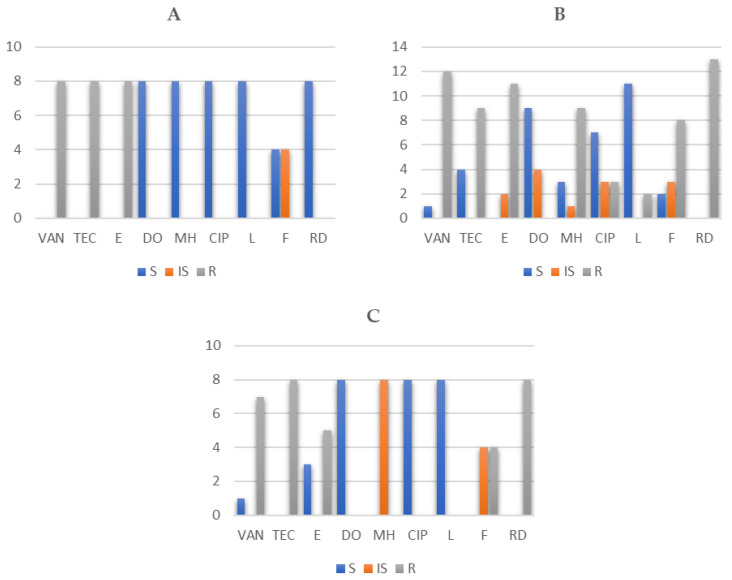
Number of resistant (R), intermediately resistant (IS), and susceptible (S) *E. faecium* (*n* = 8) (**A**), *E. faecalis* (*n* = 13) (**B**), and *E. durans* (*n* = 8) (**C**) in goat cheese samples. *n*: number strains; VAN: vancomycin; TEC: teicoplanin; E: erythromycin; DO: doxycycline; MH: minocycline; CIP: ciprofloxacin; L: levofloxacin; F: nitrofurantoin; RD: rifampicin.

**Figure 2:**
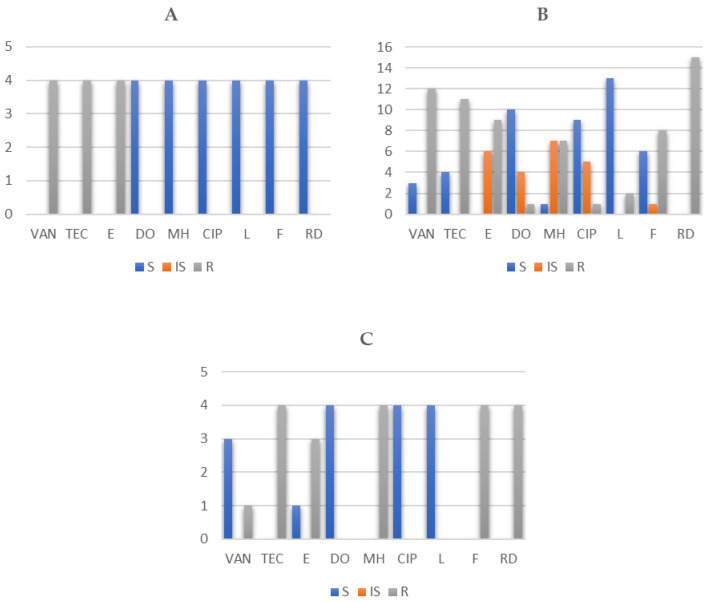
Number of resistant (R), intermediately resistant (IS), and susceptible (S) *E. faecium* (*n* = 4) (**A**), *E. faecalis* (*n* = 15) (**B**), and *E. durans* (*n* = 4) (**C**) in sheep cheese samples. *n*: number strains; VAN: vancomycin; TEC: teicoplanin; E: erythromycin; DO: doxycycline; MH: minocycline; CIP: ciprofloxacin; L: levofloxacin; F: nitrofurantoin; RD: rifampicin.

Identified isolates of *Enterococcus* sp. showed high antibiotic resistance to vancomycin (84.62%; 44/52 isolates), teicoplanin (84.62%; 44/52 isolates), erythromycin (76.92%; 40/52 isolates), and rifampicin (76.92%; 40/52 isolates). Lower antibiotic resistance was detected against nitrofurantoin (46.15%; 24/52 isolates) and minocycline (38.46%; 20/52 isolates). Individual AMR profiles of enterococci in sheep and goat cheese samples are shown in Figure 1 and Figure 2.

For the antibiotic vancomycin at a concentration of 32.0 mg/L, out of a total of 52 isolates of *Enterococcus* sp., 28 isolates (53.85%) were resistant, while at a concentration of 64.0 mg/L, 16 isolates (30.77%) were resistant. The antibiotic teicoplanin at a concentration of 32.0 mg/L showed a number of resistant isolates (30; 57.67%). At twice the concentration of the antibiotic teicoplanin 64.0 mg/L, 14 isolates were resistant (26.92%), a decrease of 53.33% compared to the dose of 32.0 mg/L. The *p* value of vancomycin and teicoplanin is significantly lower than the alpha, and this difference is statistically significant (*p* < 0.05). Resistance to the antibiotic erythromycin (E) was determined from concentrations of 8.0 mg/L and 16.0 mg/L. At a concentration of 8.0 mg/L, 31 isolates were resistant (59.62%). However, at a concentration of 16.0 mg/L, only 9 isolates (17.31%) were resistant. The odds ratio (OR) is 7.05, which demonstrates that there is a 7.05 times higher chance that an isolate shows resistance at a dose of 8.0 mg/L than at a dose of 16.0 mg/L (*p* < 0.0001). For the antibiotic nitrofurantoin (F), the MIC was set at a cut-off value of 128.0 mg/L and 256.0 mg/L. The results showed that 20 isolates (38.46%) were resistant at a concentration of 128.0 mg/L. A statistically significant decrease (*p* < 0.001) in the number of resistant isolates of enterococci (7.69%; 4) was found at a concentration of 256.0 mg/L. Rifampicin-resistant strains had an MIC determined from 4.0 to 8.0 mg/L. At a concentration of 4.0 mg/L, 29 isolates were resistant (55.77%), while at a concentration of 8.0 mg/L, 11 isolates (21.15%) were resistant (*p* < 0.001). All other antibiotics, doxycycline (DO), minocycline (MH), ciprofloxacin (CIP), and levofloxacin (L), did not show a statistically significant change (*p* > 0.05) in the number of resistant enterococci at different antibiotic concentrations (Table 1).

The occurrence of multidrug-resistant strains is of great importance in the spread of antimicrobial resistance. In a series of tests, multidrug-resistant strains were confirmed in 80% of a total of 52 strains. Specifically, multidrug resistance to five antibiotics simultaneously (VA-TEC-E-F-RD) was most frequently confirmed in *E. durans*. In species *E. faecalis*, multidrug resistance to four antibiotics simultaneously (VA-TEC-F-RD) was observed. In three strains of *E. faecalis*, multidrug resistance to eight antibiotics simultaneously was also confirmed, namely VA-TEC-E-MH-CIP-L-F-RD. In *E. faecium*, resistance to three antibiotics simultaneously (VA-TEC-E) was confirmed. 

All 40 erythromycin-resistant isolates were tested for *ermA, ermB*, *ermC*, and *msrC* genes [24,25,26,27]. Among erythromycin-resistant isolates, *ermB* (*n* = 23) was the most common resistance gene, followed by *ermA* (*n* = 9), *ermC* (*n* = 2), and *msrC* (*n* = 1), of which one *E. faecium* isolate, two *E. faecalis* isolates, and one *E. durans* isolate had *ermB* and *ermA*, and one *E. faecalis* isolate had *ermA* and *msrC*. The presence of *ermB*, *ermA*, and *ermC* was also detected in one *E. faecalis* isolate. The phenotypic expression of erythromycin resistance was confirmed in all isolates in which the presence of at least one gene encoding erythromycin resistance was confirmed (Figure S2). In addition to the detection of genes encoding erythromycin resistance, the presence of the *vanA* gene, which encodes vancomycin resistance, was detected in the test isolates. Teicoplanin resistance was also phenotypically manifested in 40 vancomycin-resistant isolates, indicating a phenotype of vanA isolates. For these isolates, the presence of the *vanA* gene was confirmed in 21 isolates by PCR. Specifically, the phenotype and genotype of *vanA* were confirmed in 6 isolates in *E. faecium* isolates, in 11 isolates in *E. faecalis*, and in 4 isolates in *E. durans*. The *ermA, ermB, ermC*, and *vanA* resistance genes were also detected in one *E. faecalis* isolate that had confirmed multidrug resistance to VA-TEC-E-MH-CIP-L-F-RD. In one *E. faecium* isolate that showed phenotypic resistance to VAN, TEC, E, *ermB*, *ermA*, and *vanA* genes were detected. At the same time, the presence of the *ermB*, *ermA*, and *vanA* genes was detected in the isolate phenotypically evaluated as multidrug resistant to VA-TEC-E-F-RD. The above multidrug-resistant isolates reported a vanA phenotype.

## 4. Discussion

*Enterococcus* species are ubiquitous bacterial. The most common include *Enterococcus faecium*, *Enterococcus durans*, and *Enterococcus faecalis* [28]. Biendo et al. [29], in turn, determined that more than half of the analyzed dairy products intended for direct consumption contained *Enterococcus bacteria*, and *E. faecium* was observed to be the predominant species. It has previously been reported that *E. faecium* (52.6%), *E. durans* (17.7%), *E. hirae* (16.4%), and *E. faecalis* (12.8%) are also common in Serbian cheeses [30]. The proportion of *E. faecium* (57%), *E. durans* (22%) and *E. faecalis* (16%) in Slovak bryndza cheese were set [31]. Jamet et al. [32] evaluated 126 samples of soft, semi-hard, and hard traditional French cheeses and observed a high prevalence of *E. faecalis* isolates (81%) compared to *E. faecium* (9.5%) and *E. durans* (7.7%) Similar results were reported by Oguntoyinbo and Okueso [33] in a study with 30 samples of traditional fermented dairy products (wara and nunu) made from unpasteurized milk in Nigeria. The occurrence of resistant *E. faecalis* in sheep’s milk has been confirmed by several studies [34,35,36]. 

The high level of resistant enterococcal contamination of the samples may be due to the actual contamination of the milk as well as the fact that these bacteria are resistant to pasteurization temperature and are resistant to various substrates and developmental conditions (temperatures, pH, salinity, etc.) [29,32]. Previous studies in Turkey have reported that the prevalence of enterococci in cheese samples ranges between 62% and 99%. The study by Sanlibaba and Senturk in 215 traditional cheese samples identified 99.1% enterococcal isolates that were highly resistant to nalidixic acid (100%), kanamycin (98.6%), and rifampicin (78.4%), and were resistant to ampicillin, ciprofloxacin, erythromycin, tetracycline, penicillin G, chloramphenicol, gentamycin, and streptomycin [37,38]. Enterococci are able to acquire antimicrobial resistance through horizontal gene transfer [39]. 

The resistance profile of *Enterococcus* species according to [36] is as follows: erythromycin (49.2%); vancomycin (37.3%); and tetracycline (45.8%). Concurrently, the detected occurrence of antibiotic resistance genes in theses tested enterococci is as follows: *ermA* 44.8%, *vanA* 63.6%, *tetA* 51.9%, *tetM* 55.6%, *ermB* 13.8%, and *vanB* 22.7%. This study may reveal that RTE food products may be reservoirs of detectable enterococci such as *E. casseliflavus*, *E. durans*, *E. hirae*, *E. gallinarum* [36]. 

Jamet et al. [32] noted in their work that enterococci may be present in cheese as a possible intermediate for the transmission of multidrug resistance. In Gaglio et al. [5], out of a total of 40 strains, resistance was highest for ERY (21 strains) and for CIP (14 strains). No resistance to penicillin, ampicillin, vancomycin, levofloxacin, linezolid, or gentamicin was observed. A total of 31 enterococci from 40 strains showed resistance to at least one antimicrobial compound. Three strains showed a multidrug-resistant phenotype (resistance to at least three antimicrobials). The study confirmed that most dairy enterococci are vectors for the spread of genes for antimicrobial resistance and virulence. Cheeses therefore represent an interesting environment for deepening studies on the risk and contribution of enterococci in fermented foods in terms of their qualified presumption of safety (QPS).

The presence of resistant enterococci in cheeses was also confirmed by our study. The antimicrobial cohesion found may vary between species of enterococci, therefore species identification of the tested isolates is required. Laser absorption/ionization matrix mass spectrometry (MALDI-TOF MS) has enabled the rapid and accurate identification of many microorganisms in the last decade [40,41].

A previous study [42] confirmed the presence of *E. durans*, *E. faecium*, and *E. faecalis* in the examined cheeses by the MALDI-TOF method, as we did in our study. 

Enterococci may pose a public health problem due to their resistance to cephalosporins, lincosamides, penicillins, and low levels of aminoglycosides [4]. Enterococci isolated from dairy products also express a similar resistant gene profile as the profile of enterococci isolated from human infections [5]. *E. durans* is a bacterium susceptible to vancomycin, ampicillin, tetracycline, chloramphenicol, and aminoglycosides [7]. However, this species is in some cases resistant to erythromycin. Resistance to erythromycin, a representative of macrodile antibiotics, is a matter of concern because macrodiles are a common substitute for individuals allergic to penicillin [43,44].

One type is associated with the mechanism of macrolide resistance by methylation of 23S rRNA, with the methylating enzyme mainly encoded by *erm* genes [45]. In a previous study [46], the *ermB* gene associated with methylation represented 96.0% of all macrolide-resistant *E. faecalis*. Out of 143 isolates, 140 were *ermB* positive.

Chajecka-Wierzchowska et al. [47] in their study confirmed a high incidence of erythromycin-resistant strains (14.3%). Resistance to macrolides was only associated with the presence of the *ermB* and *ermA* genes in individual strains. Conjugative mobile genetic elements were identified in 15.3% of strains. Regardless of the species, erythromycin testing was associated with the presence of the constitutive *ermB* gene. A high percentage of strains also contained either a combination of the *ermA* and *ermB* genes, or only the *ermA* gene [47].

In addition to testing the erythromycin susceptibility of the isolates, the study also detected resistance to vancomycin, which was confirmed in 84.7% of the total 52 isolates tested. In a study by Chajecka-Wierzchowska et al. [47], two isolated strains of *E. faecalis* were resistant to this antibiotic; high MIC values of these strains (>259 μg/mL vancomycin) are a cause for concern. Their genotypic analysis did not reveal the presence of the *vanA* or *vanB* genes; therefore, they hypothesized that vancomycin resistance may be encoded by other genes not analyzed in this study, such as *vanD*, *vane*, or *vanG*. Similar observations were made by Gomes et al. [48]. 

Historically, vancomycin-resistant isolates have been identified in the out-of-hospital region of the country, where avoparcin has been approved for use as a growth stimulator [49]. In Europe, the prevalence of vancomycin-resistant isolates from animal-based food products has been steadily declining since avoparcin was banned in animal production [50,51]. Previous studies have shown that enterococci isolated from RTE food can transmit resistance genes of vancomycin to *E. faecalis* strains. It was found that more than 70% of the tested strains were able to conjugate the transfer of at least one of the antibiotic resistance genes [47,52,53].

Similarly, in Pieniz et al. [54], in addition to vancomycin sensitivity analyses, *E. durans* was evaluated for the presence of resistance to the vancomycin gene, where the presence of the *VanA*, *vanC1*, and *vanC2* genes was not confirmed by PCR in the tested isolates. The presence of vancomycin resistance genes in strains present in animal feed is essential because the antibiotic is not metabolized by animals and therefore remains in an active form in the gut [55] that promotes vancomycin-resistant enterococci (VRE). In Europe, non-hospitalized patients, animals, and the environment are often the source of VRE (especially the *vanA* genotype) [56,57].

In the last two decades, the prevalence of VRE strains has been increasing in severe enterococcal infections that are difficult to treat. The VRE was first reported by [58]. Subsequently, the occurrence of VRE has been reported in many countries around the world. This is a particularly big problem in the Western world [59].

In a study by Perin et al. [60], no isolate presented a positive result for the *vanB* gene. In contrast, four *Enterococcus* isolates presented positive results for the *vanA* gene but tested negative according to vancomycin resistance phenotype assay. In our study of 48 isolates resistant to phenotypic vancomycin, only 21 isolates showed the presence of the *vanA* gene. This suggests that in other phenotypic VREs, resistance may be related to other typical gene resistances, such as *vanB*, *vanC*, *vanD*, *vanE*, or *vanG*. Vancomycin resistance occurs in isolates due to the transmission of genetic determinants of resistance. Vancomycin-resistant enterococci have been implicated as a common cause of nosocomial infections [61,62,63].

In our study, the vanA phenotype was most frequently confirmed based on the VRE phenotype and genotype (91.6%). *VanA* glycopeptide resistance is characterized by acquired inducible resistance to both vancomycin and teicoplanin, while the VanB phenotype is characterized by variable levels of vancomycin resistance but sensitivity to teicoplanin [64,65]. Over the last 5 years, vancomycin-resistant enterococci (VRE) with the *vanA* genotype that are susceptible to teicoplanin (i.e., having the vanB phenotype and the *vanA* genotype) have been increasingly occurring in Asia [66,67,68].

In addition to the aforementioned antibiotics, the present study focused on the detection of resistance to other antibiotics. High percentage of resistance was noted in *E. faecalis* to rifampicin (100%), vancomycin (85.7%), teicoplanin (71.4%), erythromycin (71.4%), minocycline (57.1%), nitrofurantoin (57.1%), ciprofloxacin (14.3%), and levofloxacin (14.3%). *E. durans* showed resistance to rifampicin (100%), teicoplanin (100%), vancomycin (66.7%), erythromycin (66.7%), nitrofurantoin (66.7%), and minocycline (33.3%), and *E. faecium* showed resistance to vancomycin, teicoplanin, and erythromycin (100%). In a study by Chajęcka-Wierzchowska et al., [47], the highest percentage of resistance detected among the analyzed *Enterococcus* strains was to erythromycin (14.3%), followed by tetracycline (11.6%) and rifampicin (8.7%). The percentage of strains resistant to the remaining antibiotics was determined to be from 3.2% to 1.1%, and none was teicoplanin resistant. Two strains of *E. faecalis* were resistant to vancomycin, the drug of last resort against severe Gram-positive bacterial infections (MIC > 256 mg/mL); however, no *vanA* or *vanB* genes were identified. Genes encoding vancomycin resistance have only been reported in *E. casseliflavus* and *E. gallinarum*, and these have been associated with natural bacterial vancomycin resistance encoded in the *vanC1* and *vanC2* or *C3* genes. In total, 26 (13.75%) multidrug-resistant strains and 23 different multidrug-resistant phenotypes were identified. Three *E. faecalis* isolates were resistant to linezolid, an antibiotic approved for use in 2000. Phenotypes of resistance to various drugs were observed for both *E. faecalis*, *E. faecium*, and other enterococci species isolated in the mentioned study.

The results of our study confirm the occurrence of resistant and multidrug-resistant enterococci in cheese samples. The MALDI-TOF mass spectrometry method used in the study to identify individual species of enterococci has a lower discriminant index in determining the clonality of strains compared to molecular methods, which may lead to testing of the same strains. This could be the cause of the higher degree of resistance to most of the tested antimicrobials; however, our percentage results of antimicrobial resistance of individual antibiotics correlate with the above studies.

In general, views on enterococci are diverse and ambiguous. Some countries classify them as cultures beneficial to cheese production, however, on the other hand, we can classify them as bacteria that cause health problems. Resistance to antibiotics in terms of pathogenicity, as well as the production of biogenic amines, designates enterococci among undesirable microorganisms in food [42].

## 5. Conclusions

Based on the above results, the incidence of antimicrobial resistance in enterococcal isolates from sheep and goat cheeses was determined. At the same time, the presence of genes determining resistance to erythromycin and vancomycin was confirmed in the isolates. The presence of resistant enterococci indicates the risk of the spread of antibiotic resistance in foodstuffs of animal origin in a selected part of the border area of Slovakia with Hungary.

## Figures and Tables

**Table 1:** Number of resistant^®^, intermediately susceptible (IS), and susceptible (S) species of *Enterococcus* sp.

	ATB	VAN	TEC	E	DO	MH	CIP	L	F	RD
**MIC (mg/L)**	**0.25**	-	-	3	-	-	-	-	-	-
	**0.5**	-	-	1	-	-	32	-	-	5
	**1**	-	-	2	-	-	8	27	-	7
	**2**	5	-	2	28	6	8	21	-	-
	**4**	3	4	4	15	10	3	-	-	29 ^a^
	**8**	-	4	31 ^a^	8	16	1	3	-	11 ^b^
	**16**	-	-	9 ^b^	1	11	-	1	7	-
	**32**	28 ^a^	30 ^a^	-	-	9	-	-	9	-
	**64**	16 ^b^	14 ^b^	-	-	-	-	-	12	-
	**128**	-	-	-	-	-	-	-	20 ^a^	-
	**256**	-	-	-	-	-	-	-	4 ^b^	-
	**OR**	2.6250	3.7013	7.0529	3.0583	1.2818	3.1224	3.1224	7.5000	4.6996
	**95% CI**	1.18–5.86	1.62–8.43	2.85–17.47		0.48–3.41	0.31–31.0	0.31–31.0	2.34–23.99	1.99–11.12
	***p* value**	<0.05	<0.05	<0.0001	>0.05	>0.05	>0.05	>0.05	<0.001	<0.001

^a,b^ Means within a row different superscript differ (*p* < 0.05); 95% CI: confidence interval estimate/chance of being resistant at a given ATB concentration; ATB: antibiotics; MIC: minimal inhibitory concentration; VAN: vancomycin; TEC: teicoplanin; E: erythromycin; DO: doxycycline; MH: minocycline; CIP: ciprofloxacin; L: levofloxacin; F: nitrofurantoin; RD: rifampicin. Black lines represent the breakpoints that categorize enterococci as “resistant”.

## Data Availability

The data presented in this study are available upon request from the corresponding author.

## Supplementary Materials

The following are available at https://www.mdpi.com/article/10.3390/foods10081844/s1: Table S1: Descriptions of primers used in this study; Figure S1: Workflow of detection of resistant enterococci from cheeses samples; Figure S2: Heatmap demonstration of the presence and absence of genes across species.
