# Supplementary material for: Antimicrobial Resistance of Enterococcus sp. Isolated from Sheep and Goat Cheeses
Source: Foods. 2021 Aug 10;10(8):1844. doi: 10.3390/foods10081844 (PMC8391679; doi:10.3390/foods10081844)
Supplement: Supplementary file 1 [file foods-10-01844-s001.zip › foods-1290480-supplementary.pdf]

Supplementary Materials

Table S1. Descriptions of primers used in this study.

Primer name	Primer sequence (5′-3′)	Annealing temperature (°C)	Size of PCR product	Reference
Enterococcus sp.				
Ent1	TACTGACAAACCATTCCATGATG	55	112	[19]
Ent2	AACTTCGTCACCAACGCGAAC			
**Internal control**				
InKo1	GGAGGAAGGTGGGA TGAGG	55	241	[20]
InKo2	ATGGTGTGACGGGCGGTGTG			
**Resistance**				
vanA F	TCTGCAATAGAGATAGCCGC	52	377 bp	[24]
vanA R	GGAGTAGCTATCCCAGCATT			
ermAF	TAACATCAGTACGGATATTG	54	200 bp	[25]
ermAR	CTGGATAAAATTTGGGTTGA			
ermBF	CCGAACACTAGGGTTGCTC	54	139 bp	[25]
ermBR	ATCTGGAACATCTGTGGTATG			
ermCF	TCAAAACATAATATAGATAAA	50	642 bp	[26]
ermCR	GCTAATATTGTTTAAATCGTCAAT			
msrCF	GCAAATGGTGTAGGTAAGACAACT	51	399	[26]
msrCR	ATCATGTGATGTAAACAAAAT			[27]

Figure S1

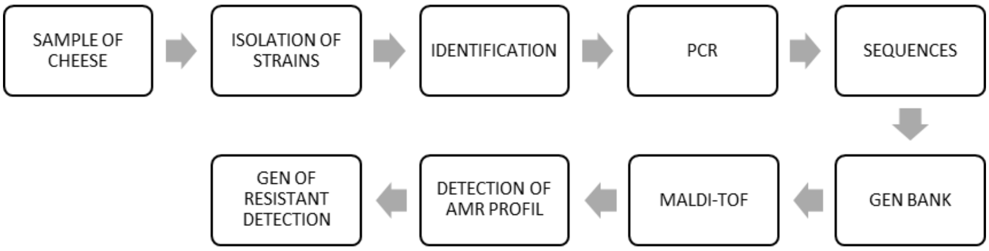

Figure S1. Workflow of detection resistant enterococci from cheeses samples.

Figure S2

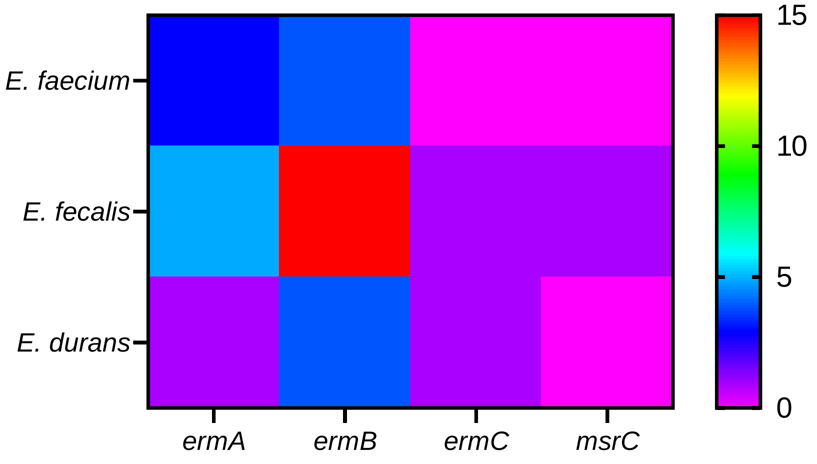

Figure S2. Heatmap demonstration of the presence and absence of genes across species.
